# Functional Homologous Recombination Assay on FFPE Specimens of Advanced High-Grade Serous Ovarian Cancer Predicts Clinical Outcomes

**DOI:** 10.1158/1078-0432.CCR-22-3156

**Published:** 2023-02-20

**Authors:** Sanna Pikkusaari, Manuela Tumiati, Anni Virtanen, Jaana Oikkonen, Yilin Li, Fernando Perez-Villatoro, Taru Muranen, Matilda Salko, Kaisa Huhtinen, Anna Kanerva, Heidi Koskela, Johanna Tapper, Riitta Koivisto-Korander, Titta Joutsiniemi, Ulla-Maija Haltia, Heini Lassus, Sampsa Hautaniemi, Anniina Färkkilä, Johanna Hynninen, Sakari Hietanen, Olli Carpén, Liisa Kauppi

**Affiliations:** 1 Research Program in Systems Oncology, Research Programs Unit, Faculty of Medicine, University of Helsinki, Helsinki, Finland.; 2 Department of Pathology, University of Helsinki and HUS Diagnostic Center, Helsinki University Hospital, Helsinki, Finland.; 3 Department of Obstetrics and Gynecology, Helsinki University Hospital, Helsinki, Finland.; 4 Department of Obstetrics and Gynecology, Turku University Hospital, Turku, Finland.; 5 iCAN digital precision cancer medicine flagship, University of Helsinki and Helsinki University Hospital, Helsinki, Finland.

## Abstract

**Purpose:**

Deficiency in homologous recombination (HR) repair of DNA damage is characteristic of many high-grade serous ovarian cancers (HGSC). It is imperative to identify patients with homologous recombination–deficient (HRD) tumors as they are most likely to benefit from platinum-based chemotherapy and PARP inhibitors (PARPi). Existing methods measure historical, not necessarily current HRD and/or require high tumor cell content, which is not achievable for many patients. We set out to develop a clinically feasible assay for identifying functionally HRD tumors that can predict clinical outcomes.

**Experimental Design:**

We quantified RAD51, a key HR protein, in immunostained formalin-fixed, paraffin-embedded (FFPE) tumor samples obtained from chemotherapy-naïve and neoadjuvant chemotherapy (NACT)-treated HGSC patients. We defined cutoffs for functional HRD separately for these sample types, classified the patients accordingly as HRD or HR-proficient, and analyzed correlations with clinical outcomes. From the same specimens, genomics-based HRD estimates (HR gene mutations, genomic signatures, and genomic scars) were also determined, and compared with functional HR (fHR) status.

**Results:**

fHR status significantly predicted several clinical outcomes, including progression-free survival (PFS) and overall survival (OS), when determined from chemo-naïve (PFS, *P* < 0.0001; OS, *P* < 0.0001) as well as NACT-treated (PFS, *P* < 0.0001; OS, *P* = 0.0033) tumor specimens. The fHR test also identified as HRD those PARPi-at-recurrence–treated patients with longer OS (*P* = 0.0188).

**Conclusions:**

We developed an fHR assay performed on routine FFPE specimens, obtained from either chemo-naïve or NACT-treated HGSC patients, that can significantly predict real-world platinum-based chemotherapy and PARPi response.

*See related commentary by Garg and Oza, p. 2957*

**Translational Relevance**

It is imperative to identify high-grade serous ovarian cancer (HGSC) patients with homologous recombination–deficient (HRD) tumors, as deficiency in homologous recombination (HR) confers sensitivity to platinum-based chemotherapy and to PARP inhibitors (PARPi). Currently used genomics-based HRD estimates have limitations and often require relatively high tumor percentage, which is rarely attained for neoadjuvant chemotherapy (NACT)-treated HGSC patients. We established a functional HR assay that can be performed on formalin-fixed, paraffin-embedded (FFPE) tumor specimens obtained from chemo-naïve as well as NACT-treated HGSC patients. Functional HRD (fHRD) status significantly correlated with favorable real-world clinical outcomes, such as longer overall survival and longer PARPi response. The functional HR test can easily be implemented in the clinical setting as immunostainings on FFPE sections are routinely performed in pathology departments; the assay does not involve time-consuming and bioinformatics-heavy DNA sequencing data analysis, and it can be performed even on samples with low tumor cell content.

## Introduction

DNA-damaging agents constitute the cornerstone of many classical cancer therapies. For high-grade serous ovarian cancer (HGSC), the most lethal gynecologic malignancy, platinum derivatives are used as DNA-damaging compounds in standard of care. Platinum intercalates with DNA, causing inter- and intrastrand cross-links, which result in single- and double-stranded DNA breaks in S phase (1, 2). These lesions are lethal to the cell if not repaired. The key DNA repair pathway for overcoming platinum-induced DNA damage is homologous recombination (HR). Accordingly, HGSCs with high HR capacity are typically platinum-resistant, while HRD ones are at least partially platinum-sensitive. Moreover, HRD tumors are also sensitive to PARP inhibitors (PARPi; refs. 3, 4). PARPi maintenance therapy has transformed HGSC management in the last few years (5, 6). Favorable outcomes of PARPi therapy strongly correlate with platinum sensitivity (7–10).

Given the markedly better response of patients with HRD tumors both to platinum and to PARPi, they ideally should be identified already at the time of diagnosis, that is, prior to starting drug treatment. Conversely, patients with HR-proficient (HRP) tumors could be channeled to alternative drug regimens. Since the advent of PARPi in clinical use, HRD testing has been a topic of high interest, and several approaches exist to identify HRD tumors (reviewed in 11–13). The two main ones include: (i) sequencing of key DNA repair genes, such as *BRCA1, BRCA2* or *PALB2*, to identify pathogenic variants, both germline and somatic (14), (ii) quantifying HRD-associated genomic features in tumor DNA (15–17), with often these two combined (18, 19). In addition, *ex vivo* functional assays of DNA damage induction and repair have been developed (20–25). Recently, detection of RAD51, a central HR protein, in immunostained formalin-fixed, paraffin-embedded (FFPE) sections without externally induced DNA damage has shown great promise in identifying HRD breast cancers (26–28). A similar approach has also been employed to gynecologic cancers, including some cases of advanced HGSC (29, 30).

In the clinic, HRD stratification currently rests on HR gene mutation testing and HRD-associated genomic features [e.g., Myriad genomic instability score (GIS)]. These genomics-based HRD tests, however, have limitations. HRD is often, but not always associated with inactivating mutations in DNA repair genes, and the functional consequences of many mutations are difficult to infer (31). Genomic scars and HRD-associated mutational signatures report on “historical”, but not necessarily current HR status of the tumor. This poses a problem because restoration of HR capacity *in vivo* occurs at appreciable frequency (32), a process that will be missed if relying on HRD genomic signatures. Furthermore, genomic scar-based HRD estimates, such as the Myriad GIS, can be determined reliably from a specimen only when tumor percentage is higher than 30%. Such tumor percentage is rarely attained for many patients with HGSC treated with neoadjuvant chemotherapy (NACT), who constitute a substantial proportion of all cases (>45%; ref. 33).

With PARPi having entered widespread clinical use as first-line maintenance therapy, there is an urgent unmet need for new and improved methods for accurately and comprehensively identifying those patients who benefit from this potent but rather costly treatment (34). Performing functional HR testing on FFPE specimens is an attractive concept, because such samples are taken routinely from each patient for histopathological analysis. Accordingly, this approach was named a priority by the ESMO Translational Research and Precision Medicine Working Group (11). Its utility for predicting real-world therapy sensitivity in advanced HGSC remains to be assessed. Importantly, thus far functional HR has not been systematically quantified from NACT-treated specimens obtained at interval debulking surgery (IDS), that is, from surgery after NACT.

The herein presented RAD51-based functional HR (fHR) test on immunostained FFPE sections quantifies real-time function of HR repair in the tumor, does not require high tumor cell content and works on both treatment-naïve and NACT-treated HGSC specimens. Thus, it can overcome many of the limitations that genomics-based methods have. An important technical discovery was that the specimens’ short time-to-fixation is critical for clinically meaningful fHRD results. We show that the fHR test predicts progression-free survival (PFS) and other key, real-world clinical outcomes in patients with HGSC, including overall survival (OS) after PARPi treatment in the recurrent setting. The fHR test provides a valuable tool to support clinical decision-making for platinum and PARPi treatment.

## Materials and Methods

### Patients

**Figure 1.**
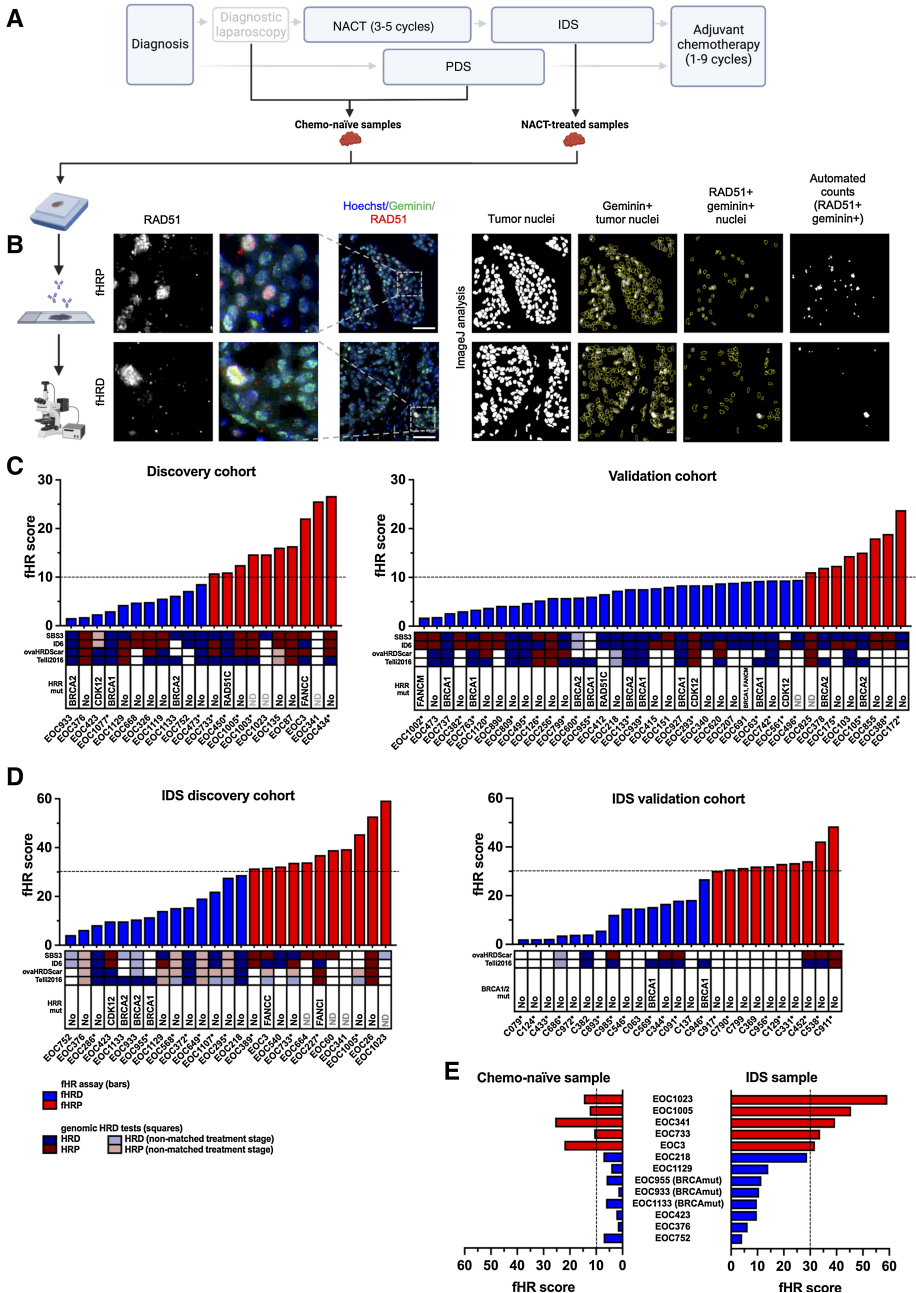
RAD51-based assay to determine fHR capacity from chemo-naïve and NACT-treated clinical HGSC specimens. **A,** Diagram showing the sample collection. Chemo-naïve samples were obtained from PDS or DL. NACT-treated specimens were obtained from IDS. **B,** Workflow of the fHR assay. Example images of geminin (green) and RAD51 (red) double stained fHRD and fHRP samples with ImageJ analysis illustration. Number of RAD51 and geminin double positive nuclei divided by the number of geminin-positive nuclei provides the fHR score. **C** and **D,** Distribution of fHR scores in chemo-naïve samples (**C**), as well as in the IDS (NACT-treated) samples (**D**), shown separately for discovery and validation cohorts. Dashed line indicates the proposed fHRD versus fHRP cutoffs. Colored squares depict HRD estimates from genomics-based assays, with blue shades corresponding to HRD and red shades to HRP. “Non-matched treatment stage” refers to cases where the genomics-based estimate of the patient was obtained from a different surgery sample (PDS/DL vs. IDS) than the fHR score. Deleterious mutations in HR genes identified from WGS/WES data are indicated for each patient. For the IDS validation cohort, only *BRCA1/2* mutational testing results from the clinic were available. Asterisks indicate patients who received bevacizumab as part of their subsequent maintenance treatment. **E,** Comparison of fHR scores from chemo-naïve and IDS (NACT-treated) samples, obtained from the same patient (*n* = 13 patients). Abbreviations: ND, no data. (**A,** Created with BioRender.com.)

Fresh tumor tissue specimens were collected from consenting patients who underwent primary debulking surgery (PDS), IDS and/or diagnostic laparoscopy (DL; Fig. 1A) for advanced HGSC at Turku University Hospital or Helsinki University Hospital. All patients were diagnosed with FIGO stage IIIB or higher HGSC. First-line chemotherapy consisted of platinum/taxane combination. The study was conducted in accordance with the ethical principles of WMA Declaration of Helsinki and approved by the ethics boards of Hospital District of Southwest Finland and Hospital District of Helsinki and Uusimaa. Written informed consent was obtained from all patients.

A total of 117 samples collected from 74 patients in Turku University Hospital were included in the study. These included both NACT-treated patients (*n* = 33) who underwent DL and/or IDS, and patients undergoing PDS (*n* = 41). 38 of these patients received bevacizumab during chemotherapy and as maintenance treatment. All patients had at least 1 year of follow-up time from diagnosis. These samples comprised the discovery and validation cohorts of chemo-naïve samples, as well as the discovery cohort of IDS samples (Table 1). The validation cohort of IDS samples consisted of 25 samples, collected from 25 patients, at the Helsinki University Hospital (Table 1). Bevacizumab was used in the treatment of 20 patients and all patients had at least six months of follow-up time from diagnosis.

**Table 1.** Clinicopathologic characteristics of patients included in the study, divided into cohorts based on sample type (chemo-naïve or IDS).

			FIGO stage				Treatment strategy		Median age at diagnosis	Cytoreduction		Primary therapy response				PARPi		
Cohort		Total	IIIB	IIIC	IVA	IVB	PDS	NACT	(range)	Complete	Suboptimal	CR	PR	SD	PD	First-line	Recurrence	Unknown
Chemo-naïve samples	Discovery	21		12	4	5	8	13	67 (41–81)	10	11	12	6	1	2	4	1	1
	Validation	40	1	26	5	8	33	7	67 (39–83)	14	26	28	12			7	16	1
IDS samples	IDS^a^ discovery	27		18	5	4	—	27	67 (55–81)	7	20	13	10	4		4	1	3
	IDS validation	25		4		21	—	25	67 (37–81)	9	16	—	—	—	—	3		

Note: Complete cytoreduction refers to no residual tumor, suboptimal to >0 mm.

Abbreviations: CR, complete response; PR, partial response; SD, stable disease; PD, progressive disease.

^a^Samples from diagnostic laparoscopies from 2 patients included in validation cohort of chemo-naïve samples and from 12 patients in discovery cohort of chemo-naïve samples (see Fig. 1E).

### Chemo-naïve samples

Chemo-naïve samples were all from Turku University Hospital and consisted of samples from 61 patients further divided into discovery and validation cohorts. The discovery cohort consisted of 21 patients and a total of 31 samples (Table 1). Eight patients belonged to the PDS treatment arm and 13 to the NACT-treatment arm. NACT-treated patients were chosen on the basis of the availability of chemo-naïve FFPE material from diagnostic laparoscopies as well as an IDS sample, which was then analyzed as part of the IDS discovery cohort. Four patients received PARPi treatment as first-line maintenance therapy, 1 patient at recurrence and PARPi treatment status of one patient (a PARPi trial participant) was unknown.

The validation cohort consisted of 40 patients and a total of 59 chemo-naïve samples (Table 1). In this cohort, we aimed to include patients with longer (>30-month) follow-up times to allow for more robust survival analysis. Of such patients, those with sufficient archival FFPE tumor material (more likely to be the case for PDS than diagnostic laparoscopies) were chosen. In addition, 6 of the PARPi-treated patients were chosen specifically to increase the number of PARPi-treated patients for subsequent analyses. Due to these selection criteria, 33 patients in the validation cohort belonged to the PDS treatment arm and seven to the NACT treatment arm. Seven patients received PARPi as first-line maintenance therapy, 16 patients at recurrence and PARPi treatment status of 1 patient (a PARPi trial participant) was unknown.

### IDS samples (NACT-treated)

IDS samples were obtained from IDS from 27 patients treated at Turku University Hospital (Table 1). Samples obtained from DL from 12 of these patients were included also in the discovery cohort, and 2 in the validation cohort of chemo-naïve samples. Four patients received PARPi as first-line maintenance therapy, 1 patient at recurrence and the PARPi treatment status of 3 patients (PARPi trial participants) was unknown. All patients had received 3 cycles of chemotherapy prior to IDS, with the exception of one, who had received 5 cycles. Time from last NACT infusion to IDS was on average 30 days (range 15–40 days). Additional IDS samples from 25 patients were obtained from Helsinki (Table 1). Three of these patients received PARPi treatment as first-line maintenance therapy.

### PARPi first-line–treated patients

Altogether, of the patients included in this study, 15 received PARPi as first-line maintenance treatment. Five of these patients were estimated to have lost either *BRCA1* or *BRCA2* function in their tumor as a consequence of somatic or inherited mutations, and a subsequent somatic loss-of heterozygosity (LOH; referred to as *BRCA*mut throughout, see Supplementary Table S1 for details). In seven patients, no *BRCA* mutations were detected by whole-genome sequencing (WGS), and they were assumed to be *BRCA* wild-type. In addition, of the patients for whom only clinical *BRCA1/2* genetic testing results were available, 2 were found to be *BRCA*mut (Supplementary Table S1).

### PARPi-at-recurrence patients

Of the patients included in this study, 17 received PARPi treatment at recurrence. Five of these patients were *BRCA*mut and 12 patients were *BRCA* wild-type, as estimated from WGS data.

### Tumor material for FFPE blocks

FFPE tumor specimens were fixed in formalin within 2 hours after surgical removal and embedded in paraffin. The short time-to-fixation of the specimen post-resection was found to be critical—samples with time-to-fixation longer than 4 hours often displayed notable tissue degeneration, as assessed from histologic slides by a trained pathologist. Also at the molecular level, we observed alterations in specimens that experienced longer times to fixation. The amount of DNA damage (Supplementary Fig. S1A) varied between fast-fixed (<2 hours) and slower-fixed (>4 hours) samples from the same tumor, and more importantly, fHR scores substantially decreased in many samples after longer time-to-fixation (Supplementary Fig. S1B). Only fast-fixed samples were included in all subsequent steps of this study.

The following anatomic tumor locations were sampled: ovary, adnexa, fallopian tube, omentum, and peritoneum. Twenty-one patients had samples available from more than one anatomic location.

### Relapse samples

For sequencing purposes only, relapse samples were collected at Turku University Hospital from ascites or surgery. Relapse samples were available for 17 patients, of whom 11 had ascites, 1 pleural fluid, and 7 surgical samples from peritoneum, bowel, lymph node, or mesenterium.

### Histology and immunofluorescence stainings

FFPE sections of tumor specimens were deparaffinized in xylene and rehydrated in decreasing concentrations of ethanol. Sections were boiled in 10 mmol/L citrate buffer, pH 6, for 20 minutes for antigen retrieval, and immunostained according to the standard procedures with primary antibodies against cytokeratin 7 (CK7, Abcam, catalog no. ab9021, RRID:AB_306947, diluted 1:500), γH2Ax (Abcam, catalog no. ab11174, RRID:AB_297813, diluted 1:1,000), geminin (Abcam, catalog no. ab104306, RRID:AB_10889692, diluted 1:200), and RAD51 (Abcam, catalog no. ab133534, RRID:AB_2722613, diluted 1:1,000) in three combinations: CK7+γH2Ax, geminin+γH2Ax, and geminin+RAD51. Sections were then incubated with fluorescently labeled secondary antibodies (diluted 1:500, donkey anti-mouse IgG-AlexaFluor 488, Thermo Fisher Scientific, catalog no. A-21202, RRID:AB_141607; and donkey anti-rabbit IgG-AlexaFluor 647, Molecular Probes, catalog no. A-31573, RRID:AB_2536183) and nuclei were counterstained with Hoechst. Slides were digitized using Pannoramic 250 FLASH II slide scanner (3DHISTECH) at 20× magnification.

### Immunoquantification and fHR scoring

For each sample, 2 to 3 regions of interest (ROI) of 3.8 mm² area in size with high tumor cell content, marked by CK7 (35, 36), and with visible DNA damage, marked by γH2Ax, were chosen for analysis based on the CK7-γH2Ax–stained section (Supplementary Fig. S2A). The ROIs (.tiff) were analyzed in ImageJ (RRID:SCR_003070) using custom macros (Fig. 1B; Supplementary Fig. S2B). In geminin-γH2Ax and geminin-RAD51 stained sections, areas with epithelial cells were identified with the help of CK7-γH2Ax stained serial section. Next, epithelial cell nuclei were identified on the basis of their size and shape, and a mask of the nuclei was created. As HR can only be performed during the S–G_2_ phase of the cell cycle, we identified epithelial nuclei positive for the S–G_2_ phase marker geminin by applying the epithelial nuclei mask to the geminin channel. Finally, we applied the geminin-positive epithelial nuclei masks to the γH2Ax and RAD51 channels, to quantify DNA damage and HR-mediated repair, respectively (Supplementary Fig. S2B). To identify RAD51 and γH2Ax-positive nuclei, a threshold and size limit was used, followed by dilate-function to merge all foci inside a single nucleus together, as individual foci cannot be reliably counted in 20x images. Particles were then automatically counted, resulting in the number of geminin-γH2Ax or geminin-RAD51 double positive nuclei per each image. From these analyses, we obtained the following quantifications per each sample: percent of S–G_2_ phase nuclei with DNA damage and percent of S–G_2_ phase nuclei undergoing HR-mediated repair i.e., fHR score. Two separate ROIs were analyzed per stained section first. If the two areas had clearly discrepant fHR scores or the values were close to the cut-off values, a third independent area was analyzed. For samples where geminin+γH2Ax double-staining was not available, geminin+RAD51 and CK7+γH2Ax stained serial sections were used to estimate the amount of DNA damage in S–G_2_ phase cells. For chemo-naïve specimens, only samples with > 10% of S–G_2_ phase cells with DNA damage were used to generate fHR scores; 97% (87/90) of chemo-naïve specimens fulfilled this criterion. For IDS specimens, only samples with >30% of S–G_2_ phase cells with DNA damage were used; 98% (51/52) of IDS specimens fulfilled this criterion. These cutoffs were established as the fHR score cannot be higher than the percent of S–G_2_ phase cells with DNA damage, so for a sample to be scored as fHRP (later determined as fHR score of ≥ 10 and ≥ 30 for chemo-naïve and IDS samples, respectively), it must contain at least that much of S–G_2_ phase DNA damage. For each sample, a minimum of 50 geminin-positive cells were analyzed. Immunofluorescence quantification of all specimens was performed blinded to *BRCA* mutation status and to data on clinical outcomes. Every .tiff image was analyzed 2 to 3 times, depending on the consistency of the result.

### Clinical parameters

Once fHR scores were determined for each patient, we analyzed them for correlations with clinical outcomes. When an fHR score was available from multiple anatomic locations from the same patient, the highest score was chosen for analyzing correlations with clinical outcomes. For both PDS and IDS patients, primary therapy response was determined after completion of adjuvant chemotherapy with RECIST 1.1 criteria (37). Platinum-free interval (PFI) was calculated from the last platinum dosage of primary chemotherapy to the first disease recurrence. PFS was calculated from diagnosis to first relapse. Because PARPi maintenance treatment in the first-line setting prolongs PFI and PFS (5), for analyses with PFI or PFS as a variable, those patients who received PARPi treatment as first-line maintenance therapy were excluded. For analyses of disease-specific OS as a variable, also patients who received PARPi therapy at recurrence were excluded. Clinical trial patients whose PARPi treatment status was unknown were excluded from PFI, PFS, and OS analyses. Response to platinum therapy in the recurrent setting (PFS2) was defined as <6 months versus >6 months, as calculated from the beginning of second-line platinum treatment to next disease progression (37, 38).

### DNA sequencing, HR gene mutations, SBS3 and ID6 signatures

Fresh-frozen samples were whole-genome sequenced and processed as described previously (bioRxiv:2022.08.30.505808). Additional WGS samples were sequenced using NovaSeq 6000 by Novogene (Novogene Co. Ltd., UK), and further whole-exome sequencing (WES) samples using HiSeq 2000 by BGI (BGI Europe A/S, Denmark) with Agilent SureSelect human all exon V5. Only samples collected at Turku University Hospital were analyzed by WGS or WES. Of 74 patients, 69 were analyzed by WGS with blood-derived matched normal samples and one without, while 4 were analyzed by WES. For 2 of the patients with WGS, only germline analysis was possible due to lack of tumor purity.

Somatic variants were called using GATK (RRID:SCR_001876; ref. 39) Mutect2. Germline variants were called from WGS normals using GATK with allele-specific filtering and joint-genotyping. Germline variant allele frequencies in tumor samples were obtained with Mutect2 forced calling. Copy-number segmentation was performed using GATK and used as input in allele-specific copy numbers and tumor purity estimation with ASCAT (40).

Variant data was queried for deleterious mutations in the following HR pathway genes: *BRCA1/2*, *BARD1*, *BRIP1*, *CHEK2*, *MRE11*, *NBN*, *PALB2*, *RAD50*, *RAD51C/D*, *CDK12*, *ATM*, and the *FANCA/B/C/D2/E/F/G/I/L/M* (41). Mutations were considered as deleterious if causing premature stop, frameshift, or altered splicing, or if classified as pathogenic/likely pathogenic in ClinVar (42) release 2022–05–28. The likelihood of mutation homogeneity in tumor or of LOH of a germline variant was estimated according to the mutation allele frequency, locus copy number, and tumor fraction in the sequenced sample.

Signatures were independently fitted for each sample using an R implementation based on SigProfilerAttribution (43) against COSMIC reference signatures v3.2 (44). Indel 6 (ID6) signature was only analyzed in WGS patients with matched normal. Single base substitutions (SBS) signatures were adjusted for trinucleotide frequency of GRCh38 (WGS) or Agilent V5 targets (WES) without the Y chromosome and the mitochondrial genome. Signatures with at least 20% ovarian cancer occurrence in COSMIC were used as starting signatures. Sequencing artifact signature SBS60 was also included for WES samples.

Thresholds for SBS3 and ID6 positivity were determined separately and based on their bimodal distributions. Samples were considered SBS3-positive if the signature contribution was greater than zero while for ID6 positivity, a threshold of 0.2796 was used. Unit length normalized indel spectra were clustered with 2-means and the threshold that maximized F1-score predicting assignment to the high microhomology deletion cluster was chosen. Each sample's signature-derived HRD status was selected from a sequenced sample with matching tissue used in fHR assay whenever possible. A minimum purity of 5% and 10% was required for a sample to be scored as SBS3/ID6-positive, or SBS3/ID6-negative, respectively. SBS3 status was determined for 92 samples from 71 patients and ID6 status for 88 samples from 68 patients.

### Genomic HRD tests

HRD causes characteristic LOH, large-scale transitions (LST), and telomeric allelic imbalances (TAI) that can be quantified and used to identify HRD tumors (16, 45). A recently optimized algorithm (called ovaHRDScar), to quantify these allelic imbalances in HGSC, was used for classification of the samples as HRD or HRP (46). A cut-off value of ≥54 for the sum of LOH, LSTs, and TAI was used for classifying a sample as HRD. Another HRD estimate (16), which also is the basis of Myriad GIS, was calculated using the program scarHRD (47); this is hereafter referred to as Telli2016 and samples with Telli2016 scores of ≥42 and/or *BRCA1/2* mutation were denoted HRD.

Tumor purity of the samples was estimated on the basis of (i) somatic copy-number profiles using the software ASCAT (ii) variant allele frequency (VAF) of the truncal mutation in gene *TP53* (*TP53*-VAF), using the formula: 2 / [(CN / *TP53*-VAF) − (CN − 2)], where CN corresponds to the absolute copy-number value estimated by ASCAT in the corresponding truncal mutation locus, and/or (ii) visual estimation from hematoxylin and eosin (H&E) sections by a trained pathologist. The highest purity value resulting from the three criteria was selected. Samples with purity below 30% were excluded from ovaHRDScar and Telli2016 results.

### Statistical analyses

Statistical analyses were performed with GraphPad Prism 9.0 software (RRID:SCR_002798). A *P* value of < 0.05 was considered significant. The chosen statistical test for each analysis is indicated in figure legends.

### Data availability

WGS data will be available through the European Genome-phenome Archive (EGAS00001006775). Other data generated in this study are available upon request from the corresponding author.

## Results

### fHR scores

We established RAD51-based fHR scores for both chemo-naïve samples and IDS samples (Table 1). A fHR score was successfully obtained from 94% of samples (134**/**143 samples). Of the nine samples for which a fHR score could not be determined, three were excluded because the amount of DNA damage in S–G_2_ phase cells did not exceed the set thresholds (>10% for chemo-naïve, >30% for NACT-treated samples), three because they did not have enough geminin-positive tumor cells (threshold of >50 cells), and another three due to failed staining or imaging. Because of these unanalyzable samples, two patients were excluded from the validation cohort of chemo-naïve specimens (*n* = 40→38) and two from the discovery cohort of IDS specimens (*n* = 27→25) from subsequent analyses with fHR score. It was possible to obtain fHR scores even from samples with less than 5% tumor content, as estimated from H&E images analyzed by a trained pathologist (Supplementary Table S2.).

The fHR test established here is similar to functional, RAD51-based HR assays described before (26–30). One key difference is that with our analysis method, S–G_2_ phase nuclei are scored as RAD51-positive or -negative whereas others quantified RAD51-positive nuclei based on individual RAD51 focus counts [≥ 5 foci per nucleus (28); ≥2 foci per nucleus (29)]. Thus, fHR scoring can be performed from digitized slides that are scanned at a low (20x) magnification without counting individual RAD51 foci; nevertheless, presence of the RAD51 foci underlies the scoring of the nuclei either as positive or negative also in our assay. In contrast to earlier work, specimens analyzed were all advanced HGSCs. Importantly, the fHR protocol was systematically employed and optimized, for the first time, for IDS (NACT-treated) samples as well (Fig. 1A and B).

### Defining fHRD versus fHRP categories for chemo-naïve specimens

In chemo-naïve specimens from patients in the discovery cohort (*n* = 21), fHR scores ranged from 1.6 to 26.7. The scores appeared to be bimodally distributed, consistent with two HR categories (Supplementary Fig. S3). The cut-off value of 10% RAD51+ geminin+ cells (that is, fHR score 10.0) was chosen to define fHRD versus fHRP in chemo-naïve specimens; this value was recently determined to be highly predictive of HRD in a cohort of >100 breast cancer patients, as assessed by PDX-based PARPi sensitivity (28). Using this cutoff, half of discovery cohort patients—and all three *BRCA*mut cases therein—fell into the fHRD category (Fig. 1C), as expected (41).

In the validation cohort (*n* = 38) fHR scores ranged from 1.8 to 23.8. When applying the 10% cut-off value for fHRD, 79% (*n* = 30) of the patients fell into the fHRD category and 21% (*n* = 8) into the fHRP category (Fig. 1C). The validation cohort included 11 *BRCA*mut patients, nine of which were categorized as fHRD and two as fHRP. The large proportion of *BRCA*mut patients (29%) explains, at least in part, the higher proportion of fHRD patients in the validation cohort compared with the discovery cohort (where only 14% of samples were *BRCA*mut).

For 21 patients, chemo-naïve samples from more than one anatomic location were analyzed. While numerical values of fHR scores varied between the locations, the fHR category (fHRD or fHRP) remained the same for all samples from the same patient, regardless of location (Supplementary Fig. S4).

### Defining fHRD versus fHRP categories for IDS specimens

Half or more of patients with HGSC undergo NACT, followed by IDS, and laparoscopic chemo-naïve samples (that is, abundant chemo-naïve tumor material) are typically not available from these patients. Thus, it is critical to also be able to estimate fHR in samples obtained at IDS. We set out to quantify fHR for this type of specimens in an IDS discovery cohort of 27 NACT-treated patients. Because no precedent of RAD51 scoring in IDS specimens exists in the literature, we first examined the staining pattern (Supplementary Fig. S5) and numerical values of fHR scores in these samples. In the discovery cohort of IDS samples, fHR scores were substantially higher than in chemo-naïve samples, ranging from 4.2 to 59.3, and the three *BRCA*mut patients in this cohort had fHR values of 9.8, 10.6, and 11.5 (Fig. 1D). Taken together, these findings indicated that the appropriate RAD51 cut-off value for fHRD in IDS specimens should be set higher than the 10% defined for chemo-naïve specimens.

To estimate a cut-off value for fHRD versus fHRP in IDS specimens, we examined their fHR score distribution. As with chemo-naïve samples, fHR values appeared to follow a bimodal distribution (Supplementary Fig. S3), based on which a cut-off value for fHRD of 30% RAD51+ geminin+ cells was chosen. Setting the cut-off value at 30% RAD51+ geminin+ cells, 56% of our IDS discovery cohort patients (14/25) fell into the fHRD category, in line with the estimated percentage of HRD tumors in HGSC (41). We note that when using the 30% cut-off value for IDS samples, the last patient with SBS3+, ID6+ and ovaHRDScar+ features (EOC218) is captured as fHRD, as are all three *BRCA*mut patients (Fig. 1D).

In the IDS validation cohort (*n* = 25), fHR scores ranged from 2.1 to 48.5. When applying the 30% cut-off value, 60% (*n* = 15) of patients fell into the fHRD category and 40% (*n* = 10) into the fHRP category (Fig. 1D); the two *BRCA*mut patients in this cohort had fHR scores of 15.4 and 26.8 and fell in the fHRD category.

### fHR status and HR gene mutations

Given that biallelic deleterious mutations in HR genes should impair fHR, one would expect that tumors with such mutations are fHRD. In this context, *BRCA*mut status is generally considered the ground truth for HRD. Altogether, our study included 18 patients with *BRCA*mut tumors (of a total *n* = 99 individual patients, chemo-naïve and IDS cohorts considered jointly), but two of these were unscorable, due to insufficient DNA damage and/or tumor cells. Fourteen of 16 *BRCA*mut tumors (88%) were scored as fHRD. The two *BRCA*mut samples scored as fHRP were tumors with somatic *BRCA2* mutations; when relapse samples of these 2 patients were analyzed by WGS, both were found to have *BRCA2* reading frame restoring mutations.

Two patients’ (EOC412 and EOC450) tumors had deleterious mutations in *RAD51C*, a well-established HR gene. EOC412 was scored as fHRD (fHR score = 6.6), but EOC450 was scored as fHRP, when using the 10% cutoff (fHR score = 11.0; Fig. 1C). This is perhaps not surprising, given that *RAD51C* depletion reduces but does not abolish RAD51 recruitment to sites of DNA damage (48, 49). This latter example illustrates a limitation of the RAD51-based fHR assay: it cannot identify all functionally HRD samples, only those with impaired RAD51 loading. The three *CDK12*mut tumors were scored as fHRD (Fig. 1C). Samples with biallelic loss of *FANCC* or *FANCI* were scored as fHRP, whereas one tumor with biallelic loss of *FANCM* (and intact *BRCA*1/2) was fHRD. (Fig. 1C and D).

### fHR and SBS3, ID6, ovaHRDScar, and Telli2016 HRD status

In addition to the fHR score, for most of the patients we had information available from at least one of the following genomics-based HRD estimates: mutational signature SBS3, mutational signature ID6 (17) ovaHRDScar (46) and/or Telli2016 (16). Note that for many of the IDS samples, it was not possible to obtain ovaHRDScar or Telli2016 score values (IDS discovery cohort in Fig. 1D) because tumor cell content was too low (see also Supplementary Table S2); most of the values shown were from laparoscopic chemo-naïve specimens from the same patient. For 40% of patients, all the available methods for HRD determination agreed with each other (Fig. 1C and D). Patients without genomic HRD-associated features were found in the fHRD category and vice versa.

### Comparison between paired chemo-naïve and IDS samples

For 13 patients, we obtained fHR scores from both pre- and post-NACT specimens (from DL and IDS, respectively), allowing us to assess whether fHR status, as determined from these two sample types, agreed with each other. We found that pre- and post-NACT samples from all 13 patients fell into the same fHR category (Fig. 1E). Although numerical fHR scores are higher in IDS samples, NACT treatment does not seem to impact fHR status (fHRD versus fHRP), as classified using the proposed cut-off values.

### Low fHR scores correlate with better primary therapy response

**Figure 2.**
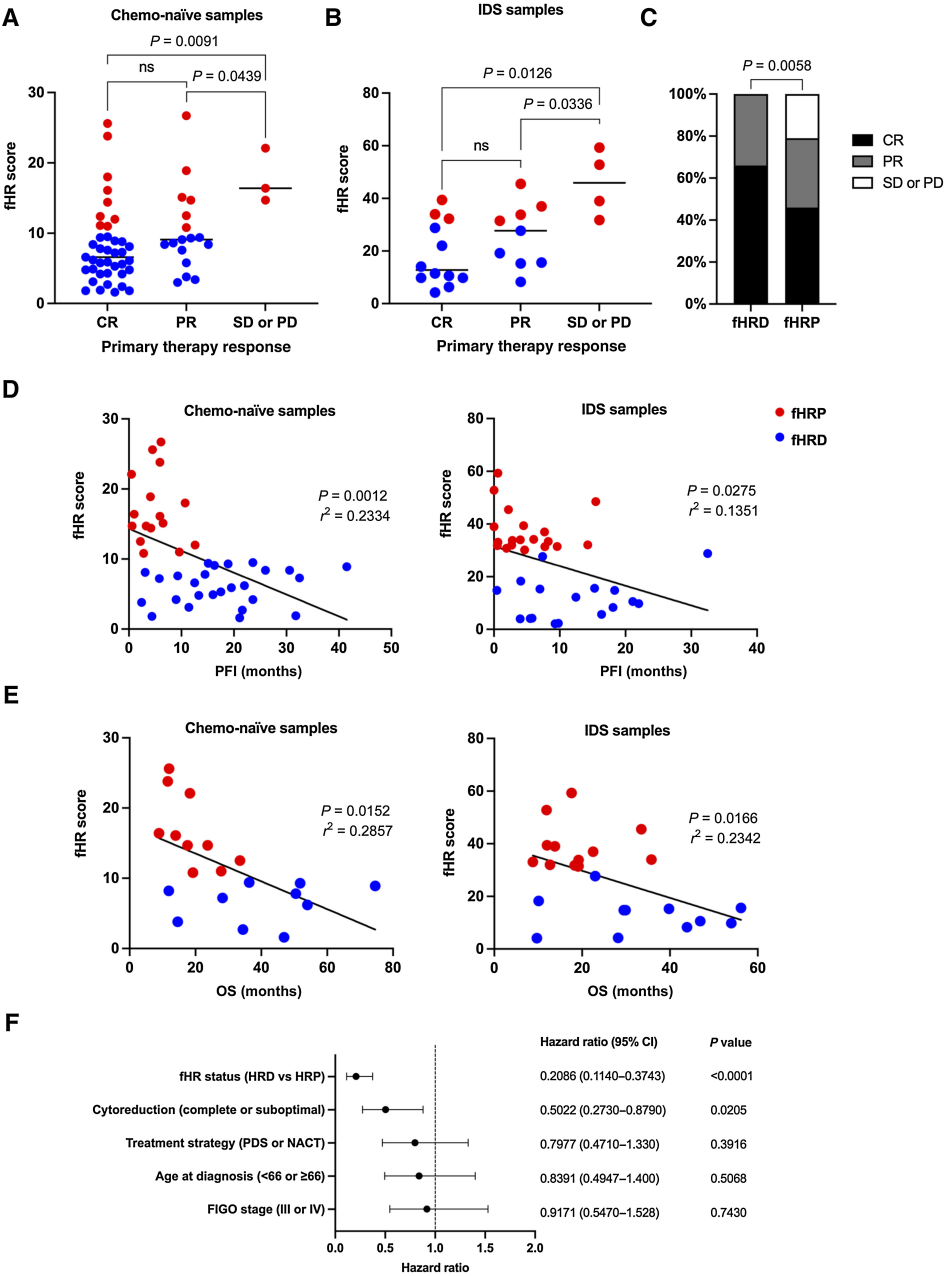
Low fHR scores correlate with better primary therapy response and with longer PFI. **A,** For patients with chemo-naïve samples, the median of fHR scores is lower in CR and PR groups compared with SD/PD (Mann–Whitney test, two-tailed). Only patients with fHRP tumors had SD/PD after primary therapy. **B,** The median of fHR scores was lower in CR and PR groups compared with SD/PD also for patients with fHR score from IDS/NACT-treated sample (Mann–Whitney test, two-tailed). **C,** Proportions of CR, PR, and SD/PD are significantly different between fHRP and fHRD groups (Fisher exact test, two-tailed). **D** and **E,** PFI and OS significantly correlate with lower fHR scores (linear regression). **F,** Multivariate hazard ratio analysis for PFI with fHR status and prognostic clinical parameters. The fHRD status and success of cytoreduction significantly correlated with longer PFI (Cox proportional hazards regression). Blue = fHRD, red = fHRP.

Platinum derivatives, used in primary therapy of HGSC, induce double-stranded breaks and thus are expected to result in better primary therapy response in patients with HRD tumors. Primary therapy response assessment was available for patients sampled at Turku University Hospital (*n* = 74). We correlated fHR scores with primary therapy response and found that low fHR scores, indicative of HRD, were enriched in complete and partial response groups. The median of fHR scores was significantly lower in patients with a complete response, compared with patients with stable or progressive disease in both chemo-naïve (6.6 vs. 16.4; Fig. 2A) and NACT-treated (12.8 vs. 45.9; Fig 2B) samples. In both groups, the median fHR score was significantly lower in the partial response group than in the stable or progressive disease group (9.1 vs. 16.4 and 27.7 vs. 45.9; Fig. 2A and B).

We then divided patients into fHRD and fHRP categories, based on their tumor's fHR score, using the cut-off values determined above. In the fHRD category, 66% of patients had a complete response to primary therapy and the rest (34%) had a partial response. In the fHRP category, 46% of patients had a complete response, 33% a partial response and 21% of patients had stable or progressive disease. The proportions of primary therapy response categories were significantly different in fHRD and fHRP categories (Fig. 2C). Surgery outcomes were similar between fHRD and fHRP groups (Supplementary Fig. S6), implying that the better primary therapy response in the fHRD group is due to better response to platinum-based chemotherapy.

### Low fHR scores correlate with longer PFI and OS

Next, we assessed whether numerical fHR scores correlated with PFI and disease-specific OS of the patients. The fHR score, obtained from either chemo-naïve or NACT-treated samples, was negatively correlated with both PFI and OS (Fig. 2D and E).

We performed a multivariate hazard ratio analysis with fHR status and clinical parameters, namely treatment strategy (PDS versus NACT), success of cytoreduction (optimal versus suboptimal), age at diagnosis (< 66 versus ≥ 66) and FIGO stage (III versus IV). Only fHR status (hazard ratio: 0.1982) and success of cytoreduction (hazard ratio: 0.5033) were found to significantly correlate with longer PFI (Fig. 2F).

### Testing alternative fHRD cut-off values

Alternative cut-off values for fHRD have been reported, e.g., 15% (29). We employed hazard ratio analysis to further test how our proposed fHRD cut-off values (10% for chemo-naïve and 30% for IDS/NACT-treated specimens), as opposed to other cut-off values, correlate with PFI. For chemo-naïve specimens, setting the cutoff to 15 or 9 also produced significant results, although the 95% confidence interval was more narrow with the cutoff of 10 (Supplementary Fig. S7). A cutoff of 25 and 35 produced significant results for IDS (NACT-treated) specimens, but again the 95% confidence interval was more narrow with the chosen cutoff of 30 (Supplementary Fig. S7). Given that all tested cutoffs produced significant results, the optimal cut-off values should be validated in larger independent HGSC cohorts with fast-fixed tumor specimens in the future.

### fHRD status correlates with PFS and disease-specific OS

**Figure 3.**
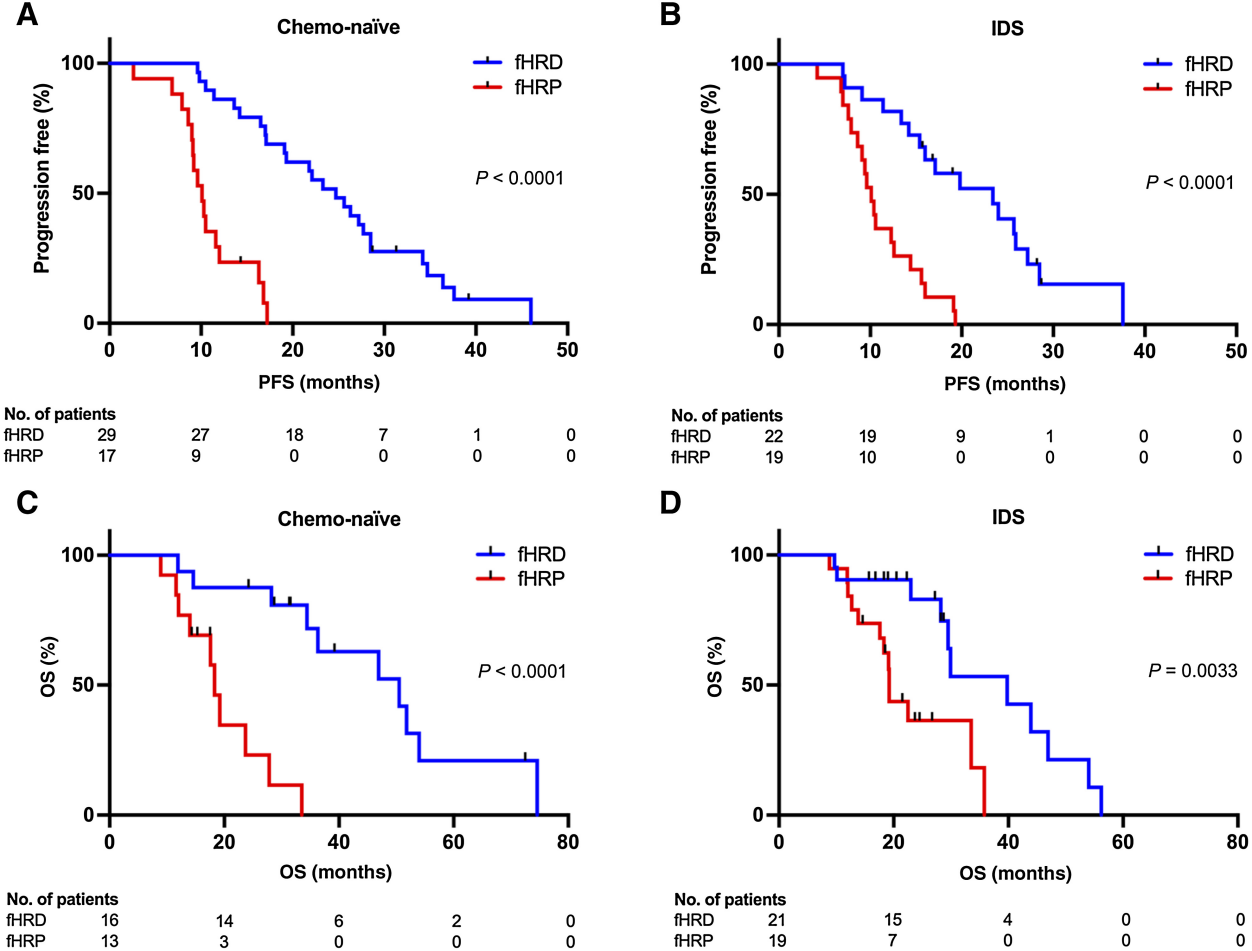
Kaplan–Meier survival analysis. fHRD status significantly predicts longer PFS and OS in chemo-naïve (**A** and **C**) and IDS (**B** and **D**) cohorts (Log-rank, Mantel–Cox test). Patients treated with PARPi in the first-line setting or with unknown PARPi treatment status were excluded from the PFS analysis (**A** and **B**). In addition to first-line and unknown PARPi treatment statuses, patients treated with PARPi at recurrence were excluded from OS analyses (**C** and **D**).

To investigate further the clinical significance of fHR status, we compared PFS of fHRD and fHRP groups in Kaplan–Meier survival analysis. The PFS-based survival curves were significantly different between the fHRD and fHRP group in both chemo-naïve (Fig. 3A) and IDS cohorts (Fig. 3B). Survival curves are shown separately for the discovery and validation cohorts in Supplementary Fig. S8A. In the chemo-naïve cohort, median PFS in the fHRD group was 24.7 months, while in the fHRP group it was only 10.1 months. The median PFS in the IDS cohort was 23.4 months in the fHRD group and 10.1 months in the fHRP group. Patients with fHRD tumors also had a better response to platinum-based chemotherapy as second-line treatment (Supplementary Fig. S8B).

To determine whether the difference in PFS between fHRD and fHRP groups was driven primarily by *BRCA*mut cases in the fHRD group, we ran the survival analysis excluding these patients. PFS-based survival curves were significantly different between the *BRCA* wild-type fHRD group (*BRCA*wt fHRD) and the fHRP group, in both chemo-naïve and IDS cohorts (Supplementary Fig. S8C). This indicates that fHRD classification—which is mutation-agnostic—captures clinically meaningful HRD that extends beyond the *BRCA*mut patient population.

In the OS analysis, survival curves were significantly different between fHRD and fHRP groups in both chemo-naïve (Fig. 3C) and IDS cohorts (Fig. 3D). In the chemo-naïve cohort, median OS was 50.5 months in the fHRD group and 18.3 months in the fHRP group. In the IDS cohort, median OS for fHRD and fHRP groups were 39.8 months and 19.2 months, respectively.

### fHRD associates with durable PARPi response

In addition to predicting platinum response, and at least as pertinent given the current HGSC therapy options, is to predict PARPi response. Of the patients analyzed here for fHR status, a subset had received PARPi maintenance treatment either in the first-line setting (*n* = 15) or at recurrence (*n* = 17). Although PARPi-treated patient numbers were small and PARPi regimens were heterogeneous, these real-world data allowed for *post hoc* assessment of how fHR status associates with *in vivo* PARPi response.

**Figure 4.**
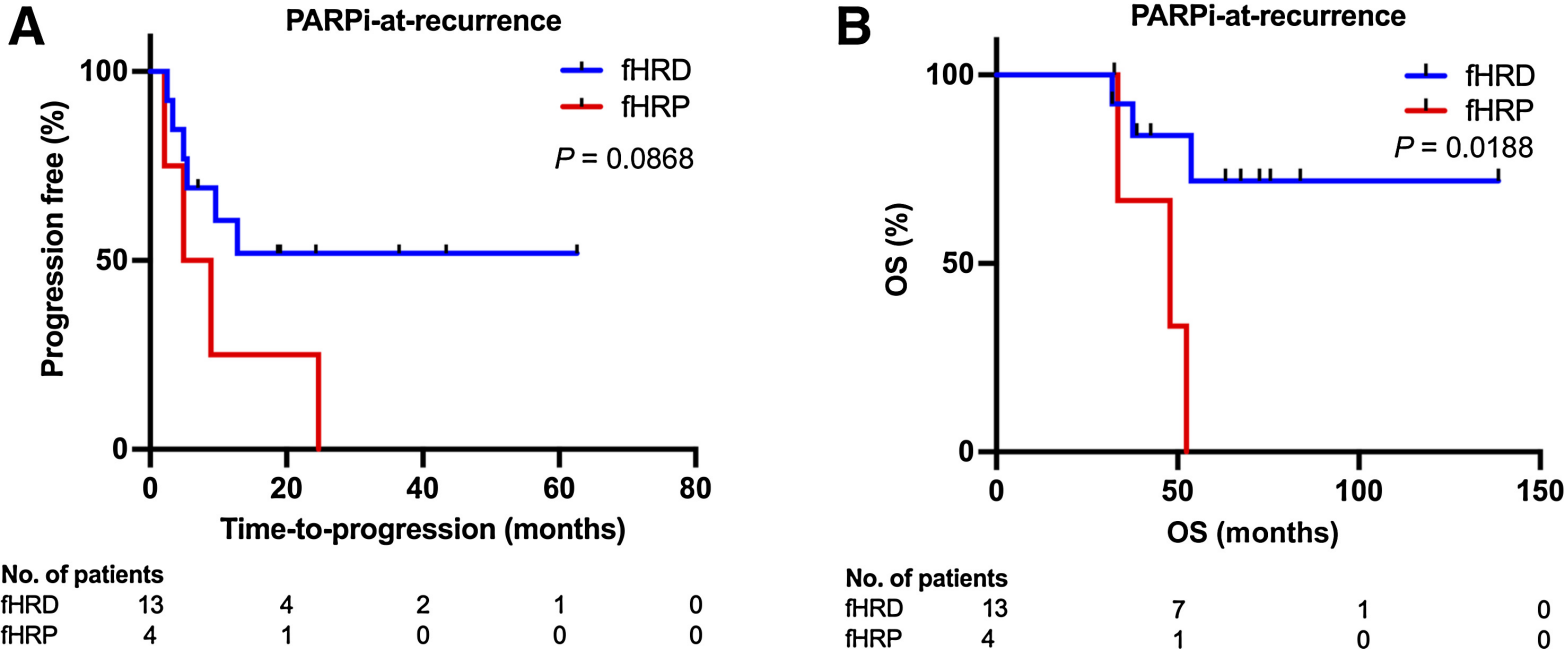
fHRD status indicates longer survival in patients treated with PARPi maintenance therapy at recurrence. **A,** In Kaplan–Meier survival analysis, time-to-progression from PARPi treatment start did not significantly differ between fHRD and fHRP patients. **B,** OS of patients in the fHRD group was significantly longer than in the fHRP group. (Log-rank, Mantel–Cox test).

In the first-line PARPi maintenance-treated cohort only two patients were fHRP, and therefore we were unable to perform any survival comparisons in this cohort. In the PARPi-at-recurrence cohort, where 4 of 17 patients were fHRP, we compared the time from the start of PARPi maintenance treatment to disease progression between fHRD and fHRP groups. Survival curves separated in favor of the fHRD group but the difference between fHRD and fHRP groups did not reach statistical significance (Fig. 4A). Notably, the fHR test was able to identify as fHRD those PARPi-treated patients with longer OS (Fig. 4B), although all 17 PARPi-treated patients were platinum-sensitive (criterion to be eligible for PARPi treatment).

### Comparison between different HRD tests

**Figure 5.**
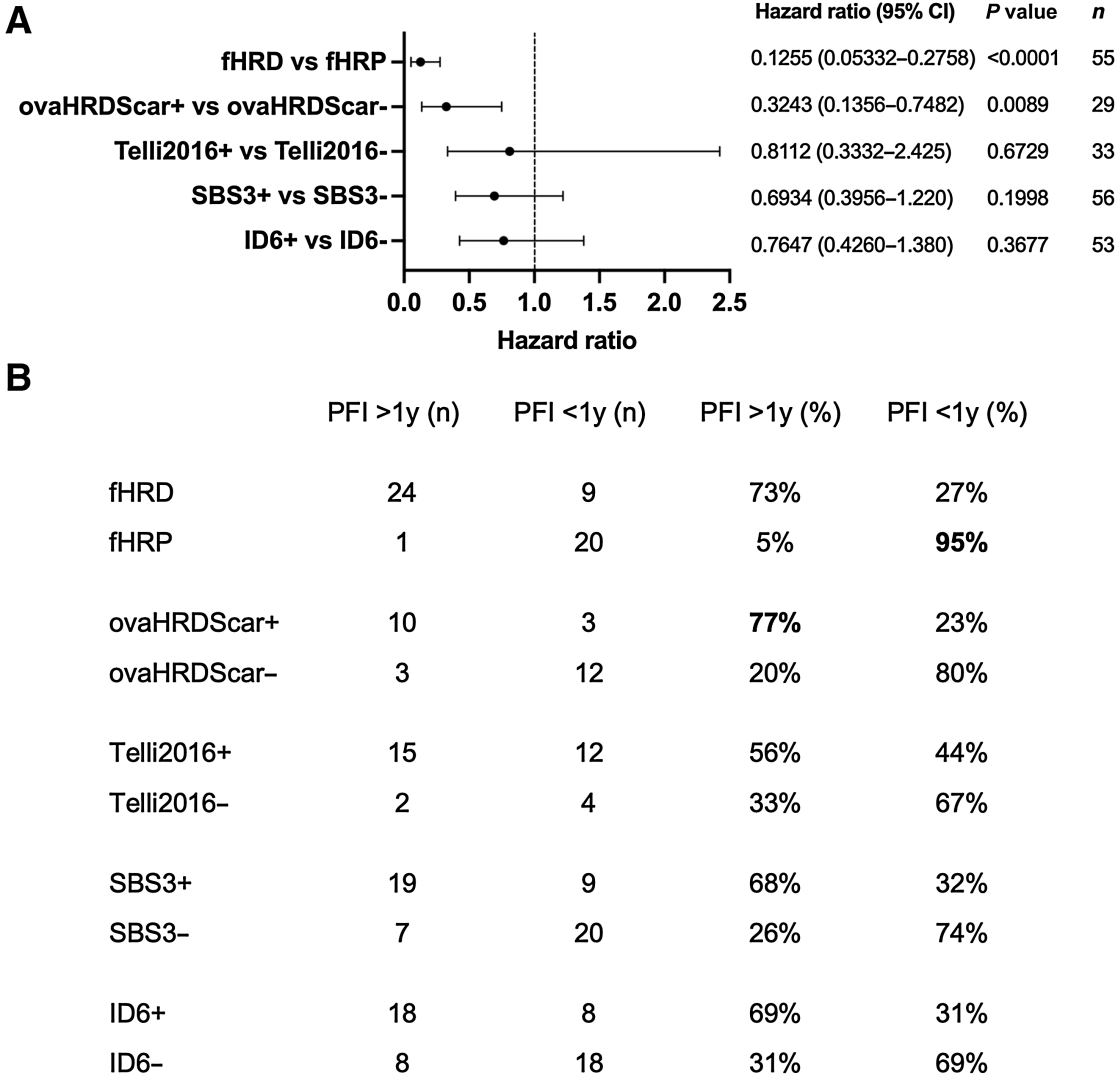
Comparison between different HRD estimates. **A,** Hazard ratio analysis with the different HRD estimates available and PFI. Only fHR and ovaHRDScar tests produced significant results (Cox proportional hazards regression). **B,** Sensitivity and specificity of the different HRD tests. ovaHRDScar has the best sensitivity in detecting clinically meaningful HRD (>1y PFI), and fHR test has the best specificity in detecting clinically meaningful HR proficiency (<1y PFI).

In addition to the fHR test result, we also had genomics-based HRD estimates—SBS3, ID6, ovaHRDScar, and/or Telli2016 HRD score—available for most patients. In hazard ratio analyses, fHR status (hazard ratio: 0.255, *P* < 0.0001) and ovaHRDScar were found to significantly correlate with longer PFI (hazard ratio: 0.3243, *P* = 0.0089; Fig. 5A). For patients with HRD tumors, as defined by fHRD, SBS3+, ovaHRDScar+, ID6+ and Telli2016+, mean PFIs were 17.5, 14.85, 18.1, 15.2 and 13.3 months, respectively. For patients with HRP tumors (fHRP, SBS3-, ovaHRDScar-, ID6-, or Telli2016-), mean PFIs were 5.2, 7.6, 6.1, 9, and 12.6 months, respectively.

We also compared the sensitivity and specificity of the different HRD tests (Fig. 5B). The ovaHRDScar test identified patients with durable response to first-line platinum-based combination chemotherapy as HRD (PFI > 1 year) with the best sensitivity (77% versus fHRD 73%, SBS3+ 68%, ID6+ 69% and Telli2016+ 56%), while fHR test identified patients with <1 year PFI as HRP with the best specificity (95% versus ovaHRDScar- 80%, SBS3- 74%, ID6- 69%, and Telli2016- 67%).

## Discussion

PARPi treatment has revolutionized HGSC treatment, but a “PARPi for all” strategy is not viable in the long term, due to high cost and appreciable side effects (34). Not all patients respond to platinum-based therapy or PARPi and should be channeled for other drug treatments. Thus, it is critical to identify patients with HRD tumors, to target the right therapy to the right patients. Assaying RAD51 function, rather than HRD-associated genomics, is an attractive approach, as it measures current, not historical, HR capacity of tumor cells and is mutation-agnostic. FFPE-based RAD51 assays have previously been described in breast cancer samples (26–28), epithelial ovarian cancer (30) and ovarian/endometrial cancer sample cohorts (29), but never for advanced HGSC specifically. No published studies exist on fHR testing from IDS samples, which constitute an appreciable fraction of available HGSC specimens. Moreover, very limited data exist (22, 25, 30) on the predictive value of fHR scores for *in vivo* therapy response in patients with advanced HGSC. In prior studies, *ex vivo* irradiation was often used to induce DNA damage before RAD51 quantification (20–25). In contrast, our method relies on endogenous DNA damage, making it easier to incorporate into the clinical workflow. Here, we show that RAD51 quantification in FFPE sections (the fHR test) of patients with unselected HGSC can identify fHRD beyond *BRCA*mut tumors and significantly predict chemotherapy response and other key clinical outcomes. Importantly, we also established a novel fHR scoring protocol for diagnostic IDS samples that predicts clinical responses to HGSC therapy. Further, the fHR test shows promise for identifying patients who benefit most from PARPi maintenance treatment. Our findings need to be validated in larger cohorts in the future, and the utility of fHR testing should be systematically assessed in first-line PARPi-treated patients.

To our knowledge, this is the first demonstration that fHR status from IDS specimens, i.e., samples that have been exposed *in vivo* to a full course of standard-of-care NACT treatment, significantly predicts therapy response, as measured by different clinical outcomes. Previously, the Vreeswijk group determined HR capacity using an *ex vivo* irradiation protocol in a cohort consisting of PDS and IDS specimens, but no significant correlation with PFI or OS was found, nor were results from IDS samples reported separately (25). Our results demonstrate that for NACT-treated IDS samples the proposed 30% cut-off value for fHRD significantly predicts two key clinical outcomes, PFS and OS. Its robustness should be tested in larger, independent cohorts of NACT-treated patients.

The results reported here are, to our knowledge, the first to show the predictive power of fHRD—as measured from routine HGSC FFPE specimens—for real-world, clinical *in vivo* platinum and PARPi responses. An added benefit of our analysis method is that it does not include counting individual RAD51 foci per nucleus, but instead defines each nucleus as RAD51-positive or negative, making the assay more robust and circumventing the need for confocal microscopy. Another major strength of the fHR assay is that it can be performed on tumor samples with a low number of proliferating tumor cells. This is critical, as NACT-treated patients who have good platinum response often have very few proliferating tumor cells left when they undergo IDS surgery. These patients are precisely those most likely to derive significant benefit from PARPi, but with existing methods (genomics-based clinical HRD tests), their tumors are often unscorable as these assays require relatively high tumor percentage.

Naturally, the fHR assay is not without limitations: it can identify only those functionally HRD samples with impaired RAD51 loading. Also, a sample must have sufficient endogenous DNA damage in S–G_2_ phase cells (>10% for chemo-naïve, >30% for NACT-treated) to generate a reliable fHR score. Of our HGSC samples, however, only three (2.1%) were excluded for too-low DNA damage in S–G_2_ phase. Thus, it seems that the vast majority of routine HGSC samples have enough endogenous DNA damage to obtain a fHR score.

Two of 16 *BRCA*mut tumors were scored as fHRP (EOC105 and EOC378) and perhaps coincidentally, both cases had somatic *BRCA2* mutations, and both were found, by WGS, to have a reversion mutation in their relapse sample. Our functional HR test, performed on the primary tumor samples, already categorized these patients as fHRP, possibly due to the presence of HRP (*BRCA2*-revertant) subclones. EOC105 had a PFI of 6.5 months, and PFI of EOC378 was 12.6 months—both clearly below the average PFI of *BRCA*mut fHRD patients (18.5 months) in our study cohort. It should be noted that not all *BRCA*mut cases behave clinically as expected. For instance, in the SOLO1 trial, which only included *BRCA*mut patients, approximately 10% relapsed within 12 months despite PARPi maintenance therapy, and nearly 50% in the placebo group failed to show a durable (>12 month) platinum response (50). It follows that one should be cautious about *BRCA*mut status as the ground truth for HRD. Ultimately, the ground truth that is most relevant in the context of HRD testing is real-world clinical response to platinum and/or PARPi.

Further developing the fHR test for relapse samples would be of high clinical interest, as mechanisms of acquired resistance are more common in this type of samples. Measurements of "real time” fHR status of tumors at relapse, rather than relying on genomics or the fHR status of the primary tumor, would help to accurately stratify patients for future treatment. We speculate that even small radiologically guided biopsies may contain enough tumor material to successfully perform fHR testing, but this needs to be assessed in future studies.

All HRD tests are plagued by some degree of false negative and false positive HRD calls. For example, genomically HRD patients—as defined by *BRCA*mut status and/or Myriad GIS—were not significantly enriched in long-term PARPi responders in the recurrent setting (51). Arguably, the most pertinent task is to better identify the *BRCA*wt HRD patient population, given that many long- and intermediate-term PARPi responders are *BRCA*wt (52). To this end, our results demonstrate that the fHR test significantly predicts long-term response to platinum-based chemotherapy (a reasonable proxy for PARPi response), even when *BRCA*mut cases were removed from analyses.

Although the number of PARPi maintenance-treated patients in our cohort was limited, this report is, to our knowledge, the first to correlate fHR status with *in vivo* PARPi maintenance response in patients with HGSC. This retrospective analysis indicates that the fHR test may be particularly useful for identifying patients who, in the recurrent setting, failed to benefit from PARPi (those with fHRP tumors). We do not have data to address the utility of fHR score in predicting response to first-line PARPi and future studies should focus on these patients.

Further strengthening the idea that fHRP status reports on HRP-like clinical behavior is the fact that fHRP patients displayed significantly worse platinum responses, both to first- and second-line treatment. Recently Hoppe and colleagues reported that high RAD51 nuclear expression scores associate with worse platinum response in ovarian cancer (30). Although in the fHR score, RAD51 nuclear positivity relies on the presence of nuclear RAD51 foci (rather than simply RAD51 nuclear expression), our finding and that of Hoppe and colleagues (30) both indicate that RAD51 is a meaningful biomarker for poorer chemotherapy outcome in HGSC. fHRP patients could in the future be channeled for alternative treatments, at least in the recurrent setting, as they are not likely to respond to platinum-based chemotherapy or to PARPi.

Our findings provide a strong motivation for including fHR testing into prospective PARPi clinical trials, and/or retrospectively analyzing archival FFPE blocks from PARPi trials. Note, however, that short time-to-fixation (<2 hours) is needed for successful fHR testing, which limits its utility of many archival FFPE samples. A failure rate of 30% was observed in a similar RAD51-based assay (53); there authors also speculate that this may be due to time-to-fixation affecting sample quality. The fHR test, as reported here, can be performed on routine FFPE samples, and has several additional practical advantages: it does not matter at which type of surgery the specimen is acquired (laparoscopic, PDS, and IDS), nor from which anatomic location.

In summary, fHRD testing shows great promise as a universal method for stratifying patients with advanced HGSC as platinum- and PARPi-sensitive. Tumor fHRD status significantly predicts clinical outcomes (both PFS and OS), as well as real-world PARPi sensitivity. We propose it should be incorporated into the HRD testing toolkit to support clinical decision-making, especially for patients for whom only a NACT-treated sample is available.

## Supplementary Material

Supplementary Table S2 Tumor percentages estimated from H&E images and comparison between HR, ovaHRDScar and Telli2016 status.

Supplementary Figure S1 The effects of long time-to-fixation on DNA damage markers

Supplementary Figure S2 Image analysis workflow in ImageJ.

Supplementary Figure S3 Binned distributions of fHR scores in the discovery cohorts.

Supplementary Figure S4 Comparisons of fHR scores obtained from different anatomical locations.

Supplementary Figure S5. Example images of staining patterns.

Supplementary Figure S6 Comparison of surgery outcome between fHRD and fHRP groups.

Supplementary Figure S7 Comparison of different fHR score cut-off values.

Supplementary Figure S8 A. PFS-based survival curves shown separately for discovery and validation cohorts. B. Response to second-line platinum therapy. C. PFS-based survival curves excluding BRCAmut fHRD patients.

Supplementary Table S1 HRR gene mutations found in WGS/WES or clinical BRCA1/2 mutational testing.
