# Supplementary material for: Functional Homologous Recombination Assay on FFPE Specimens of Advanced High-Grade Serous Ovarian Cancer Predicts Clinical Outcomes
Source: Clin Cancer Res. 2023 Feb 20;29(16):3110–23. doi: 10.1158/1078-0432.CCR-22-3156 (PMC10425726; doi:10.1158/1078-0432.CCR-22-3156)
Supplement: Supplementary Figure S1 — The effects of long time-to-fixation on DNA damage markers [file ccr-22-3156_supplementary_figure_s1_suppfs1.pdf]

Supplementary figure S1.

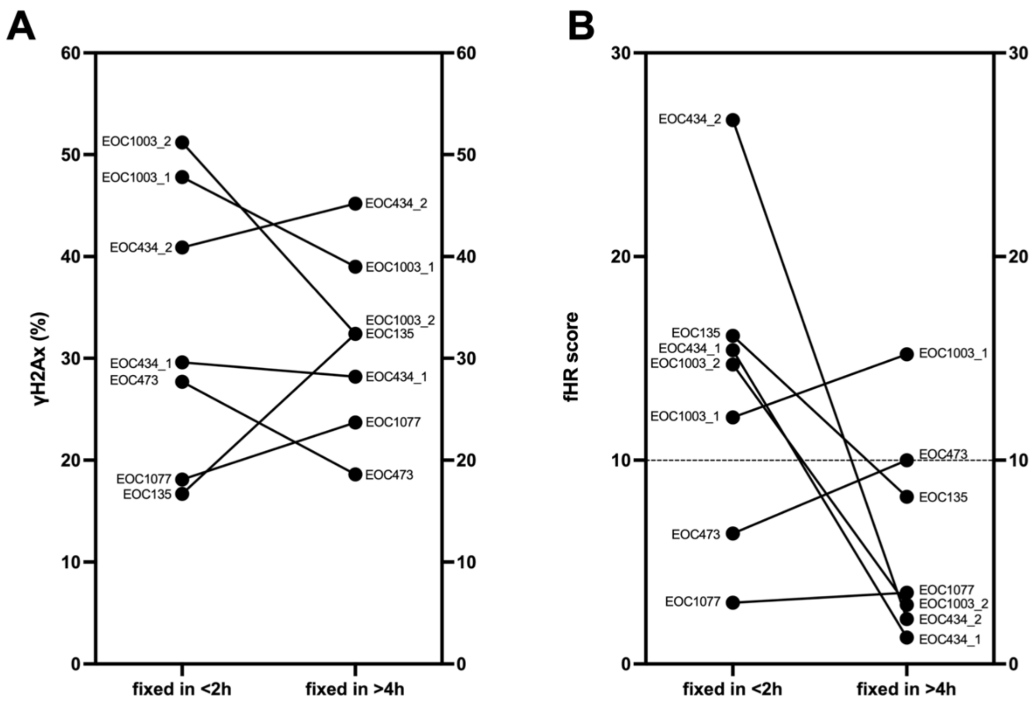

***Supplementary figure 1. Time-to-fixation of HGSC tumor specimens can alter γH2Ax positivity and fHR score**. Two pieces of the same (chemo-naïve) tumors, n=7, were fixed within <2 hours and >4 hours after surgical resection. **A.** Amount of DNA damage, marked by γH2Ax. **B.** Functional HR score analyzed from the paired samples with <2h to fixation and >4h to fixation. Dashed line indicates fHRD cut-off value (10% for chemo-naïve tumors). Four out of seven samples display a substantial drop in fHR score upon extended time-to-fixation.*
