# Supplementary material for: Functional Homologous Recombination Assay on FFPE Specimens of Advanced High-Grade Serous Ovarian Cancer Predicts Clinical Outcomes
Source: Clin Cancer Res. 2023 Feb 20;29(16):3110–23. doi: 10.1158/1078-0432.CCR-22-3156 (PMC10425726; doi:10.1158/1078-0432.CCR-22-3156)
Supplement: Supplementary Figure S2 — Image analysis workflow in ImageJ. [file ccr-22-3156_supplementary_figure_s2_suppfs2.pdf]

Supplementary figure S2.

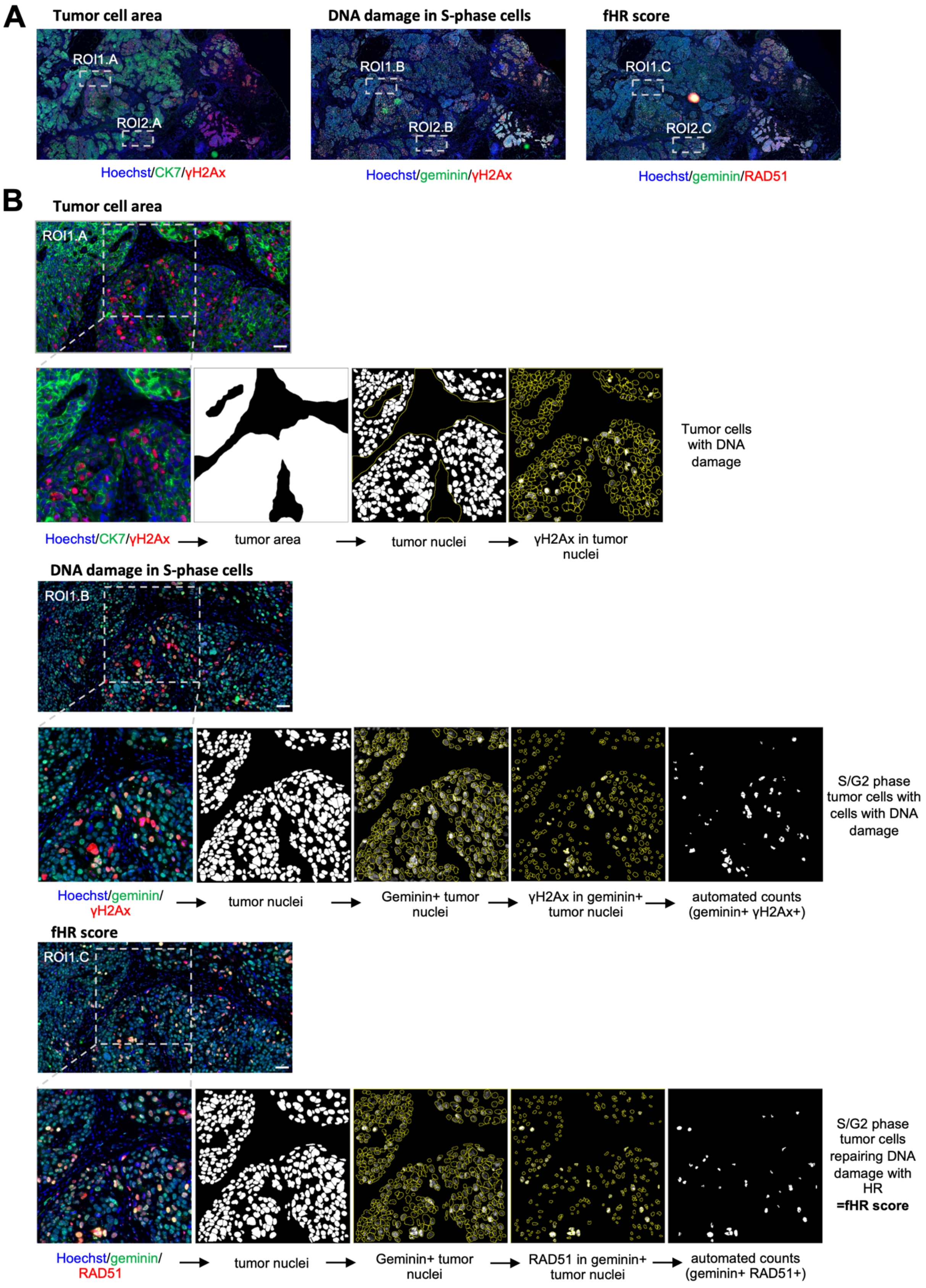

Supplementary figure S2. Area selection and image analysis workflow in ImageJ*. *A. *From CK7-γH2Ax double stained section, tumor cell areas with DNA damage are identified (ROI1&2.A). From the geminin-γH2Ax double stained section, the amount of DNA damage in S/G2 phase cells is quantified (ROI1&2.B). Functional HR score is calculated as the percentage of RAD51-geminin double positive nuclei out of all geminin positive nuclei from the geminin-RAD51 double stained section (ROI1&2.C). Two separate ROIs, each with an area of 3.8mm², were analyzed per stained section first. If the two areas had clearly discrepant fHR scores or the values were close to the cut-off values (10% ± 1 for chemo-naïve, 30% ± 2 NACT-treated samples), a third independent area was analyzed.* ***B.*** *In geminin-γH2Ax (ROI1.B) and geminin-RAD51 (ROI1.C) stained sections, areas with epithelial cells were identified with the help of CK7-γH2Ax (ROI1.A) stained serial section. A mask of the nuclei was created and applied to the geminin channel to identify epithelial nuclei in S/G2 phase of the cell cycle. Next, geminin-positive epithelial nuclei mask was applied to the γH2Ax (ROI1.B) and RAD51 (ROI1.C) channels, to quantify DNA damage and HR-mediated repair, respectively. Scale bar 50μm.*
