# Supplementary material for: Functional Homologous Recombination Assay on FFPE Specimens of Advanced High-Grade Serous Ovarian Cancer Predicts Clinical Outcomes
Source: Clin Cancer Res. 2023 Feb 20;29(16):3110–23. doi: 10.1158/1078-0432.CCR-22-3156 (PMC10425726; doi:10.1158/1078-0432.CCR-22-3156)
Supplement: Supplementary Figure S3 — Binned distributions of fHR scores in the discovery cohorts. [file ccr-22-3156_supplementary_figure_s3_suppfs3.pdf]

Supplementary figure S3.

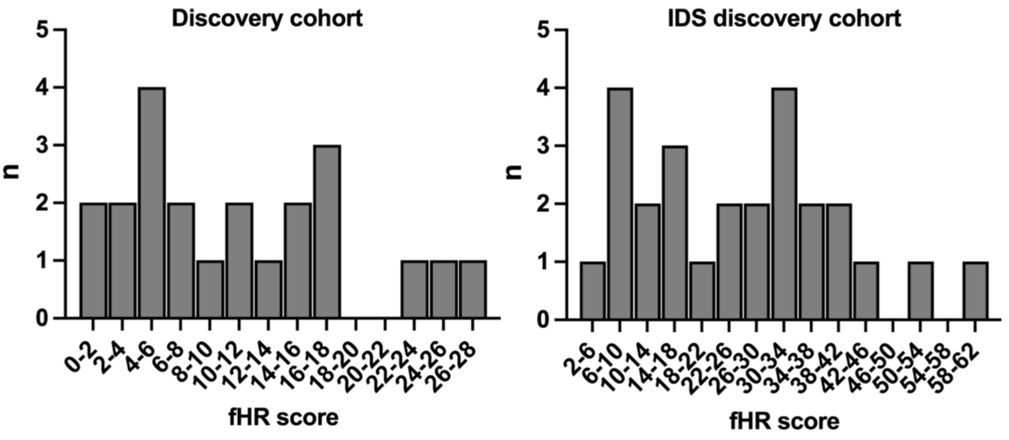

Supplementary figure S3. *Distributions of fHR scores in chemo-naïve and IDS discovery cohorts.*
