# Supplementary material for: Functional Homologous Recombination Assay on FFPE Specimens of Advanced High-Grade Serous Ovarian Cancer Predicts Clinical Outcomes
Source: Clin Cancer Res. 2023 Feb 20;29(16):3110–23. doi: 10.1158/1078-0432.CCR-22-3156 (PMC10425726; doi:10.1158/1078-0432.CCR-22-3156)
Supplement: Supplementary Figure S4 — Comparisons of fHR scores obtained from different anatomical locations. [file ccr-22-3156_supplementary_figure_s4_suppfs4.pdf]

Supplementary figure S4.

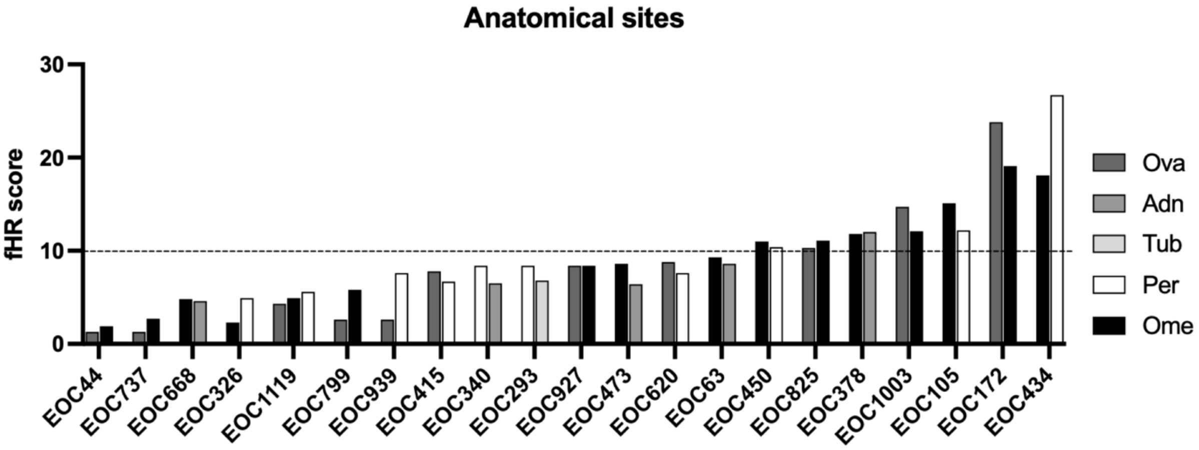

Supplementary figure S4. *Comparison between fHR scores from chemo-naïve samples obtained from different anatomical locations of individual patients. With the proposed cut-off of 10 (indicated as dashed line), fHR status of each patient remained unchanged regardless of anatomical site sampled. If a patient has an fHR score near the cut-off value, one might consider assaying other tumor sites, if feasible, in order to increase the confidence of fHR classification. Abbreviations: Ova, ovary; Adn, adnexa; Tub, fallopian tube; Per, peritoneum; Ome, omentum.*
