# Supplementary material for: Functional Homologous Recombination Assay on FFPE Specimens of Advanced High-Grade Serous Ovarian Cancer Predicts Clinical Outcomes
Source: Clin Cancer Res. 2023 Feb 20;29(16):3110–23. doi: 10.1158/1078-0432.CCR-22-3156 (PMC10425726; doi:10.1158/1078-0432.CCR-22-3156)
Supplement: Supplementary Figure S5. — Example images of staining patterns. [file ccr-22-3156_supplementary_figure_s5.suppfs5.pdf]

# Supplementary figure S5.

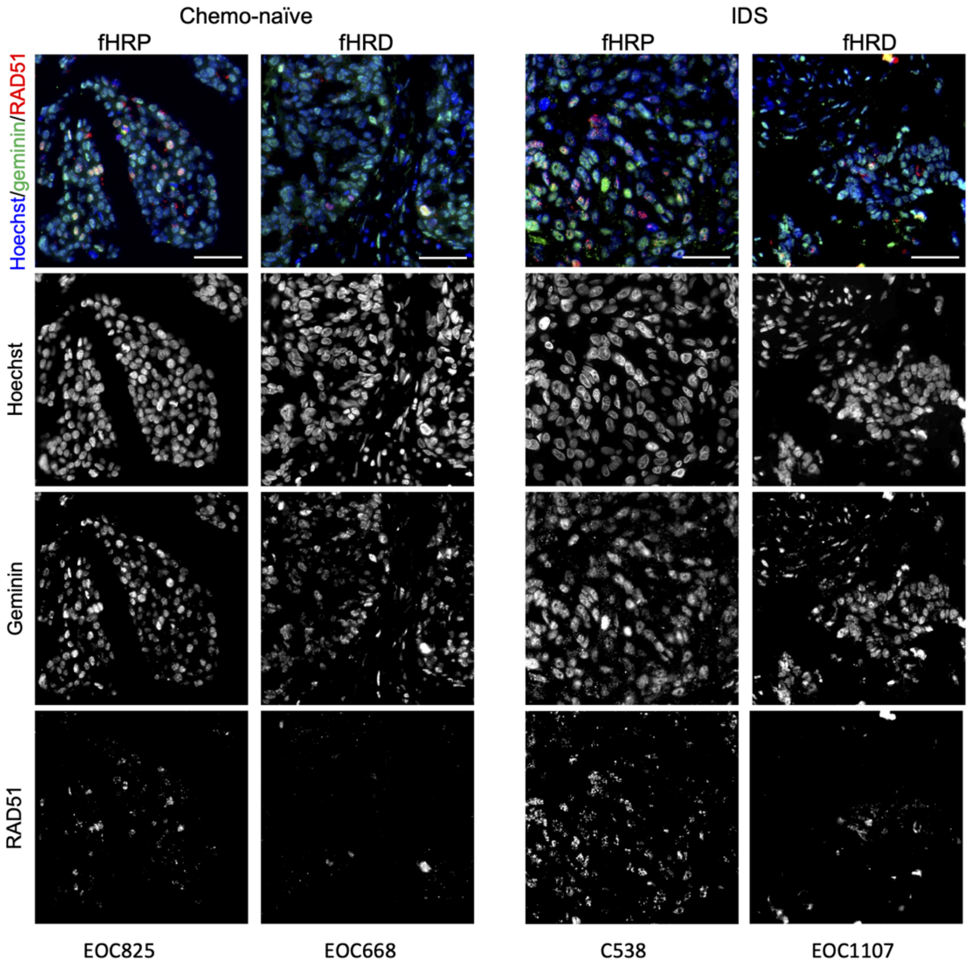


***Supplementary figure S5.*** *Example images of staining patterns in chemo-naïve and IDS (NACT-treated) geminin-RAD51 double stained specimens classified as fHRD or fHRP. Scale bar 50µm.*
