# Supplementary material for: Functional Homologous Recombination Assay on FFPE Specimens of Advanced High-Grade Serous Ovarian Cancer Predicts Clinical Outcomes
Source: Clin Cancer Res. 2023 Feb 20;29(16):3110–23. doi: 10.1158/1078-0432.CCR-22-3156 (PMC10425726; doi:10.1158/1078-0432.CCR-22-3156)
Supplement: Supplementary Figure S6 — Comparison of surgery outcome between fHRD and fHRP groups. [file ccr-22-3156_supplementary_figure_s6_suppfs6.pdf]

Supplementary figure S6.

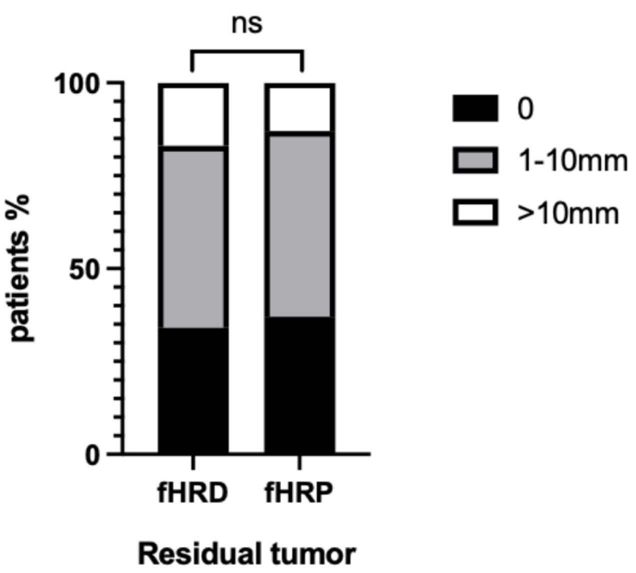

Supplementary figure S6. *Comparison between the success of cytoreduction in fHRD and fHRP groups (Chi-squared test).*
