# Supplementary material for: Functional Homologous Recombination Assay on FFPE Specimens of Advanced High-Grade Serous Ovarian Cancer Predicts Clinical Outcomes
Source: Clin Cancer Res. 2023 Feb 20;29(16):3110–23. doi: 10.1158/1078-0432.CCR-22-3156 (PMC10425726; doi:10.1158/1078-0432.CCR-22-3156)
Supplement: Supplementary Figure S7 — Comparison of different fHR score cut-off values. [file ccr-22-3156_supplementary_figure_s7_suppfs7.pdf]

Supplementary figure S7.

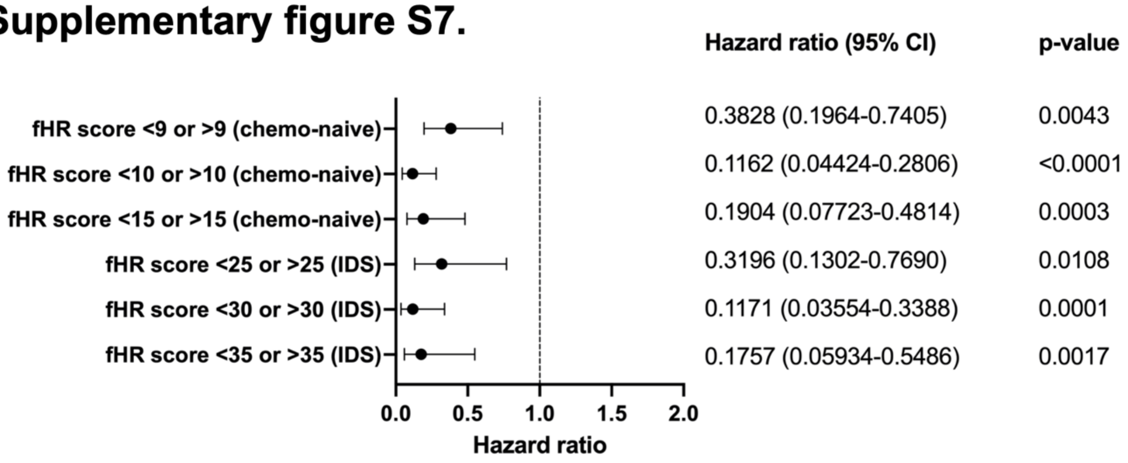

Supplementary figure S7. *Hazard ratio analysis with different cut-off values for fHR score and PFI (Cox proportional hazard regression).*
