# Supplementary material for: Functional Homologous Recombination Assay on FFPE Specimens of Advanced High-Grade Serous Ovarian Cancer Predicts Clinical Outcomes
Source: Clin Cancer Res. 2023 Feb 20;29(16):3110–23. doi: 10.1158/1078-0432.CCR-22-3156 (PMC10425726; doi:10.1158/1078-0432.CCR-22-3156)
Supplement: Supplementary Figure S8 — A. PFS-based survival curves shown separately for discovery and validation cohorts. B. Response to second-line platinum therapy. C. PFS-based survival curves excluding BRCAmut fHRD patients. [file ccr-22-3156_supplementary_figure_s8_suppfs8.pdf]

Supplementary figure S8.

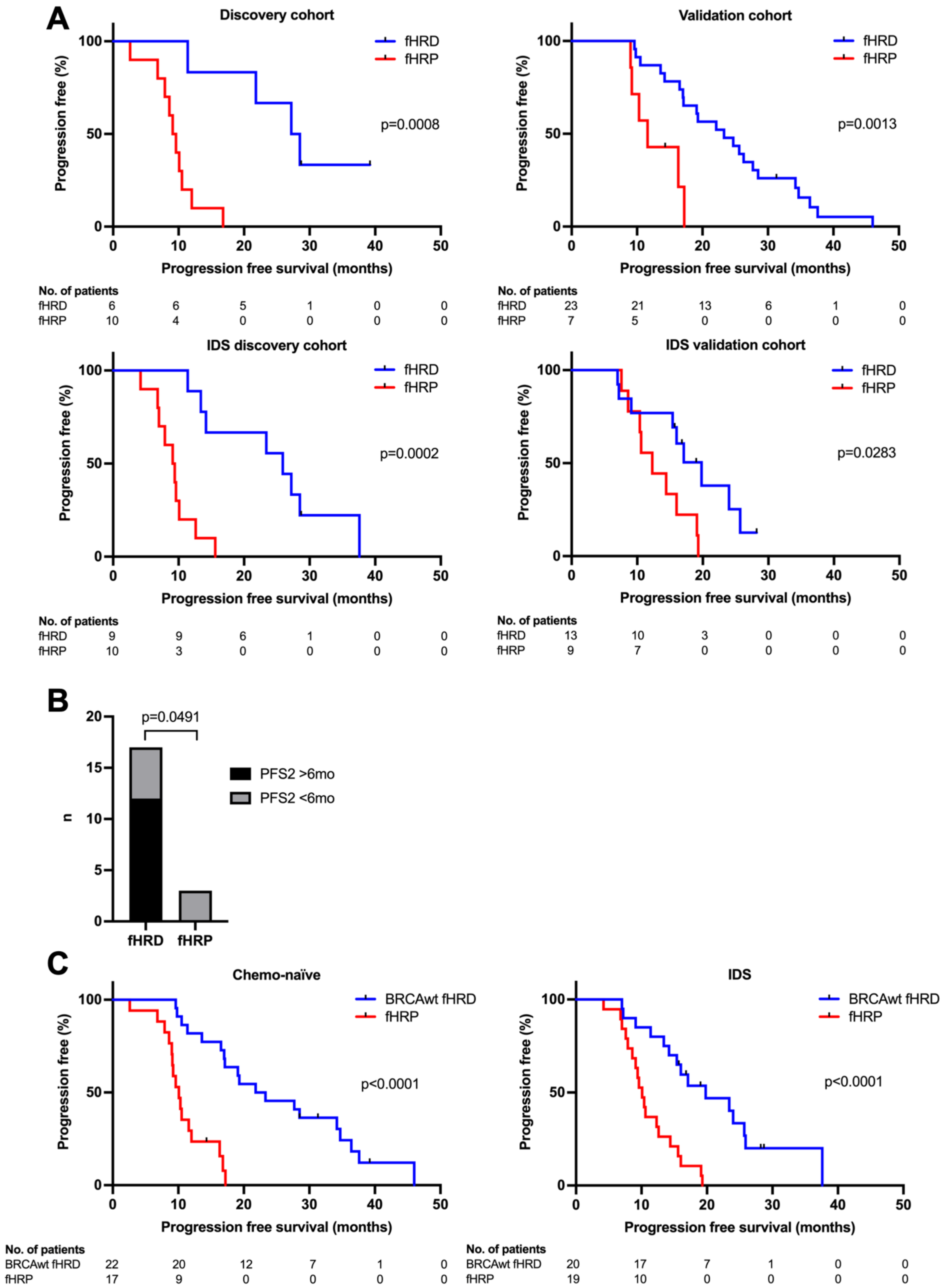

***Supplementary figure S8. Progression free survival in the different cohorts**. **A.** PFS shown separately in discovery and validation cohorts of chemo-naïve specimens, as well as discovery and validation cohorts of IDS specimens (Log-rank, Mantel-Cox test). **B.** Response to second-line platinum treatment in fHRD (n=17) and fHRP (n=3) groups (Fisher's exact). **C.** PFS in chemo-naïve and IDS cohorts without BRCAmut fHRD patients (Log-rank, Mantel-Cox test).*
